# TmaDB: a repository for tissue microarray data

**DOI:** 10.1186/1471-2105-6-218

**Published:** 2005-09-01

**Authors:** Archana Sharma-Oates, Philip Quirke, David R Westhead

**Affiliations:** 1 Academic Unit of Pathology, University of Leeds, Leeds, LS1 3EX, UK; 2 School of Biochemistry & Microbiology, University of Leeds, Leeds, LS2 9JT, UK

## Abstract

**Background:**

Tissue microarray (TMA) technology has been developed to facilitate large, genome-scale molecular pathology studies. This technique provides a high-throughput method for analyzing a large cohort of clinical specimens in a single experiment thereby permitting the parallel analysis of molecular alterations (at the DNA, RNA, or protein level) in thousands of tissue specimens. As a vast quantity of data can be generated in a single TMA experiment a systematic approach is required for the storage and analysis of such data.

**Description:**

To analyse TMA output a relational database (known as TmaDB) has been developed to collate all aspects of information relating to TMAs. These data include the TMA construction protocol, experimental protocol and results from the various immunocytological and histochemical staining experiments including the scanned images for each of the TMA cores. Furthermore the database contains pathological information associated with each of the specimens on the TMA slide, the location of the various TMAs and the individual specimen blocks (from which cores were taken) in the laboratory and their current status i.e. if they can be sectioned into further slides or if they are exhausted. TmaDB has been designed to incorporate and extend many of the published common data elements and the XML format for TMA experiments and is therefore compatible with the TMA data exchange specifications developed by the Association for Pathology Informatics community. Finally the design of the database is made flexible such that TMA experiments from several types of cancer can be stored in a single database, which incorporates the national minimum data set required for pathology reports supported by the Royal College of Pathologists (UK).

**Conclusion:**

TmaDB will provide a comprehensive repository for TMA data such that a large number of results from the numerous immunostaining experiments can be efficiently compared for each of the TMA cores. This will allow a systematic, large-scale comparison of tumour samples to facilitate the identification of gene products of clinical importance such as therapeutic or prognostic markers. In addition this work will contribute to the establishment of a standard for reporting TMA data analogous to MIAME in the description of microarray data.

## Background

**Figure 1:**
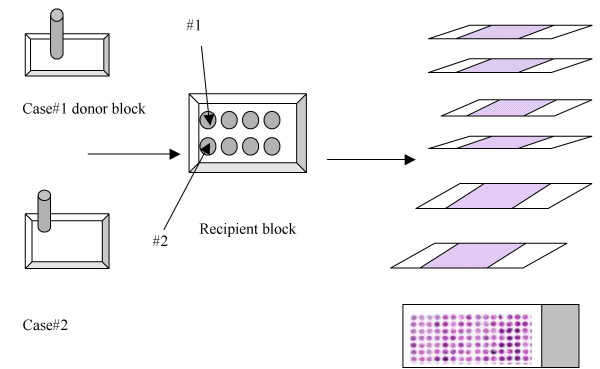
Schematic representation of the construction of a TMA block. TMAs are assembled by extracting cylindrical cores (using a 0.6 mm needle) from specific locations in the paraffin-embedded tissue blocks "donor" and re-embedded in an empty paraffin block "recipient" [2]. The TMA block is then sectioned off onto slides.

Tissue microarray technology provides a high-throughput method for large-scale immunohistochemical analyses under uniform experimental conditions. The TMA technology was developed to assist genome-scale molecular pathology studies and facilitate the analysis of molecular alterations in thousands of tissue specimens in parallel at the DNA, RNA, or protein level [1]. TMAs are constructed by drilling 0.6 mm cylindrical cores at the site of interest from various paraffin-embedded specimen blocks, referred to as donor blocks, and re-embedding them into an empty paraffin block, referred to as recipient block (Figure 1).

On a single TMA block up to 1000 specimens can be arrayed representing a number of different pathologies and tissue types both, normal and tumour. The recipient block is then sectioned off into a number of slides (from 50 – 400) [3]. These slides can then be screened at the DNA, RNA and protein level by fluorescence *in situ *hybridisation (FISH), *in situ *hybridisation (ISH), and immunohistochemistry (IHC), respectively.

There is a large quantity of data associated with a TMA experiment. These include images, quantified experimental results, patient demographics and pathology reports of each specimen [4]. The vast quantity of data generated by TMA experiments and the different types of information associated with each experiment has led to the need for a central data repository. Thus far there have only been three papers published on approaches for the archiving and analysis of TMA data and all three have limitations [5,6]. The relational database management system of Manley *et al. *[5] associates pathology data with immunohistochemical staining results but is restricted to just one type of cancer in this case being prostate cancer and cannot be adapted to include pathology data from different types of cancer. Liu *et al. *[6] on the other hand focus on the archiving and the analysis of TMA staining results but offer no means to relate the pathology information. Finally, the TAD database developed by Coombes *et al. *[7] is a tool focusing on enabling pathologists to solely score cores on TMA slides generated from a single TMA block. It is therefore considered too restrictive as a database for archiving TMA data.

Currently there are no central databases that facilitate the storage and analysis of TMA data that can also accommodate pathological information from different types of cancer. This has led to the design and implementation of TmaDB a relational database that archives all aspects of TMA data into a single database that can be accessed from remote locations to both update and retrieve data. The database design is flexible, thus incorporating pathological information from different types of cancer into one central database. The database design is compliant with recently recommended data exchange specifications [4,8].

### Construction and contents

**Figure 2:**
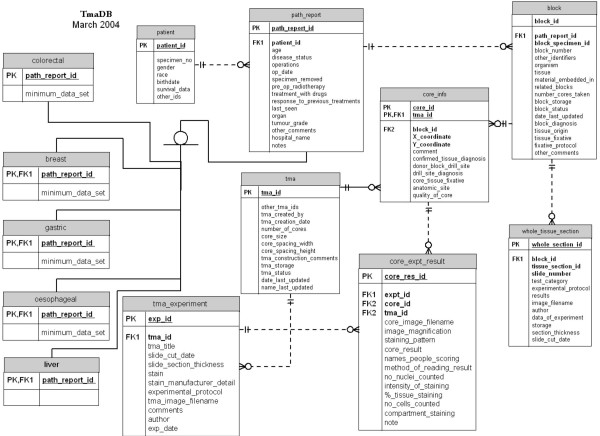
Entity relation (ER) diagram of TmaDB.

The relational database is implemented in MySQL server version 14.5. The database schema was designed by Entity-Relationship (ER) modelling (Figure 2) and can be found on the TmaDB Web site.

The "block" table is the key table in the database to which all other tables are related (directly or indirectly). This table contains the specimen identifier and other data relating to a particular specimen. The "path_report" table is another important table that stores some generic information about a patients' medical history regardless of the specific cancer diagnosis. The specific information associated with a particular type of cancer is stored in the disease-specific tables. The disease specific information is obtained from the national minimum data set required for the reporting of cancer pathological investigations. This minimum data set is a standard supported by the Royal College of Pathologists. The "tma" table stores the information about the design and the construction of the TMA block (also referred as the recipient block), which can then be sectioned onto several slides (Figure 1). Each of these slides is stained with a specific antibody or a histochemical stain. The staining protocol, the slide image and other details of the experiment are stored in the "tma_experiment" table. Each of the cores on the TMA slide has a stain result and a scanned image associated with it that is stored in the "core_expt_results" table. Other information regarding the quality of the core and the tissue diagnosis is stored in the "core_info" table. The "whole_tissue_section" table stores the staining protocol as well as the results and the images of the stain generated from the traditional whole tissue section (if available) for the clinical specimens used in the TMA.

### Content

**Figure 3:**
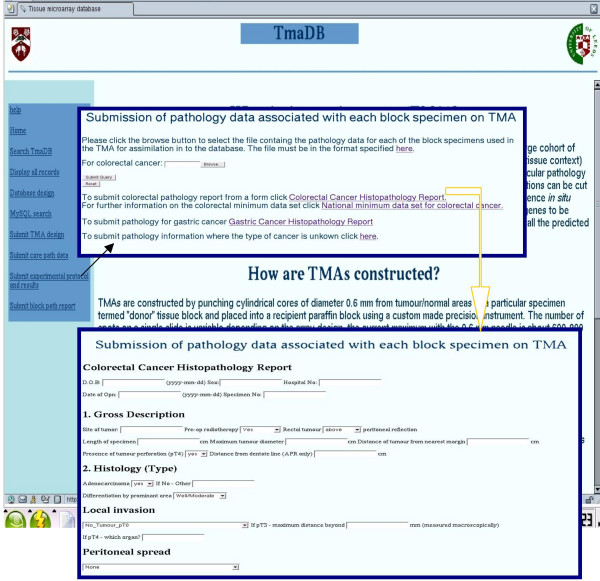
TmaDB homepage.

The data submitted to the database comes from a number of different sources. One of the data sources is the pathology report for each clinical specimen, which is sent with the specimen either directly from the hospital, or provided as part of the clinical trials. These reports are usually provided as paper copies in the form of a letter. If sent as a paper copy, they are digitised by an individual who then extracts the information that would be required for the national minimum data set for each of the specific types of cancer (Figure 3). The national minimum data set for reporting specific types of cancer is supported by the Royal College of Pathologists and the forms can be downloaded from the Royal College of Pathologists website . This information can be entered directly on the Web to be assimilated into the database or saved as a tab delimited text file that can be uploaded from the Web site. Most of the information obtained is stored in the "path_report", "patient" and "block" tables (Figure 2). It should be noted that patients' names and addresses are not stored in the database.

Information on the storage location in the laboratory of each block and whether the block is still in use is stored in the block table along with when this information was last updated. This enables a block to be found easily in the event it is required for future experiments. This information is uploaded from the tab delimited text file containing details of TMA construct design. The information recorded in this file is shown on the Web site, and can be downloaded and used as a template for recording data alongside experimental work.

The pathological details of each of the cores together with additional information such as the location on the original block from which the core was obtained and the quality of the core are uploaded from a tab delimited text file and stored in the core location table. The TMA slide immunohistological staining protocol and the results are also recorded in a tab delimited text file, an example of which is shown as a template on the Web site.

Each TMA slide is scanned using software that provides a very high-resolution image of the TMA slide enabling users to "zoom in" and "zoom out" on the image over the web. TMA slide images are saved on an image server. They can be divided into a grid such that each core image can be saved as a separate image. The TmaDB has a link to the image server for each of the cores and results from the analysis of the images are stored in the "core_expt_result" table (Figure 2). Only the filenames of the images are stored by TmaDB and the actual images are stored on the image server.

Currently there are 18 TMA experiments stored in the database although this number is set to rise. The number of people whose clinical specimens have been analysed on TMA is 1095 (April 2005). Most of this data has been generated as part of clinical trials.

## Utility and discussion

**Figure 4:**
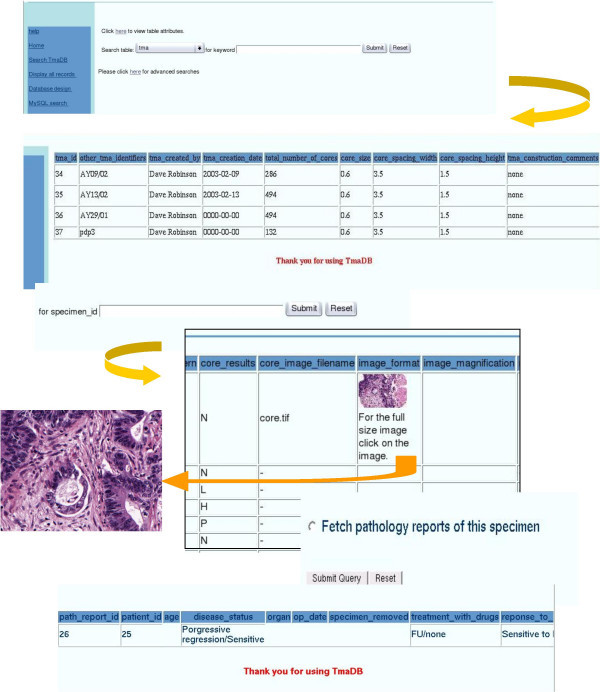
An example query of the database.

The MySQL relational database is interfaced with the World Wide Web making the database user-friendly as well as enabling access from remote locations for multiple users at any time. The web site also enables users to submit and retrieve data from the database. The homepage provides a brief background on TMA technology and outlines the advantages and disadvantages of the technique, it also provides a menu for easy navigation of the web site. A help page provides detailed explanations for each of the menu links. A brief description is given below:

The search page allows users to retrieve data from the database by searching with a specific keyword. Specific searches can be performed for cases where the "specimen_id" is already known by the user (Figure 4).

The "Display all records" link connects to a page containing all the tables in the database. Clicking on individual table names fetches all the data contained in the particular table. The "Database design" link, displays the ER diagram (Figure 2). The "MySQL search" link is an html form that enables the user to retrieve data from the database with MySQL "select" statements. For security reasons, the user only has permission to search the database but not write to the database. However badly constructed MySQL "select" statements with incorrect syntax will not be executed and the MySQL error message will be displayed on screen. In addition, the provision of SQL select facilities could leave the database vulnerable to badly constructed queries that would be damaging to performance but not data, although this has not been a problem to date. The other four links on the menu are for inputting data into the database. The "Submit TMA design" link allows the user to upload a tab delimited text file which is then saved into a directory on the server side and an email is sent automatically to the curator who then checks the file to make sure there are no errors and then the information is loaded into the database automatically. The data is loaded sequentially into the database beginning with the TMA design ("Submit TMA design") then the specific details of each of the cores on the TMA ("Submit core path data") followed by experimental protocol and results ("Submit experimental protocol and results") and finally the pathological data ("Submit block path report"). The file formats for each of the files to be uploaded are shown on the Web site. The final link on the menu connects to the image server where all images for all TMAs are archived. All files can also be uploaded in XML file format (the schema for this is provided on the Web site). In addition the output from all queries to the database can be displayed or downloaded as an XML file. The XML file format is compatible with the data exchange specification developed by Berman *et al. *[4] in that we have adopted their extensibility mechanism and therefore our instance documents are still valid under their exchange specifications. The basic XML structure developed by Berman et al. [4] has been extended with the aim of establishing a community standard that is similar to MAGE-ML for microarray databases [9].

The purpose of a central cancer TMA database is to provide a single repository for laboratory experimentalists to store TMA experiments. Storage of all TMA experiments will enable researchers to make correlations between the intensity of staining of a specific antibody and the stage of cancer, or between different types of cancers. The curation of a database will also encourage researchers to report results in a standard format thereby enabling comparisons between experiments performed by different individuals. An additional advantage of the database is to be able to determine where a specimen block is stored or the person/institution in possession of a specimen block of interest for further experiments. Furthermore it is anticipated that the database will also be used as a learning tool for pathologists.

Further developments of the database include the addition of other information from different experiments (such as cDNA microarray) on the same clinical specimen used in the TMA experiment and allowing users to carry out complex queries (see below).

An example of a complex query to the database:

Find all cases where specimens are stained with antibody "p53" AND type of cancer is "colon" AND pT stage is "3".

## Conclusion

The TmaDB provides a central repository for archiving all aspects of TMA data. The relational database includes the vast majority of the published Common Data Elements for a TMA experiment. This will therefore enable efficient data exchange as well as contributing to the establishment of a standard for reporting TMA data analogous to MIAME in the description of microarray data [10]. The database design is adaptable such that pathological data from several different types of cancer can be included in one database.

It is anticipated that in addition to providing a resource for archiving and querying TMA data TmaDB will also enable large-scale analyses of TMA data.

## Availability and requirements

Tmadb web site: .

The database and the software to upload files and query the database are available under the GPL as a package for installation on a local server [see Additional files].

## Authors' contributions

ASO participated in the design, implementation of the study and drafted the manuscript. PQ conceived of the study. DRW participated in the design and coordination of the study. All authors read and approved the final manuscript.

## Supplementary Material

Additional File 1 This compressed (gz) file contains two directories tmadb_bmc_html and tmadb_bmc and two files, create_tmadb.txt and a README file which can be extracted using gunzip software. The create_tmadb.txt file contains all the MySQL create commands for creating tables contained in the database. The README file provides instructions to help the user install the software. The tmadb_bmc_html directory contains html, xml and text files required for interfacing with the cgi programs. The tmadb_bmc directory contains ten files, nine files with the extension cgi and a file named config.pl. **config.pl **Contains variables that require modification during installation. **colo_form_input.cgi **Program to upload colorectal pathology information from the Web form. **colo_path_input.cgi **Program to upload colorectal pathology information from the Web. **core_path.cgi **Program to upload specific information relating to each core from the Web. **keysearch.cgi **Program to query the database using a keyword search or a specific specimen identifier. **mysql_search.cgi **Program to query the database using MySQL statements. **table_contents.cgi **Program to display the contents of each table in the database. **tma_construct.cgi **Program to upload TMA design construct information from the Web. **tma_result_input.cgi **Program to upload TMA experiment protocol and results from the Web. **unknown_path.cgi **Program to upload pathology information from the Web for specimens where the diagnosis is unknown. Click here for file
