# Supplementary material for: TmaDB: a repository for tissue microarray data
Source: BMC Bioinformatics. 2005 Sep 1;6:218. doi: 10.1186/1471-2105-6-218 (PMC1215475; doi:10.1186/1471-2105-6-218)
Supplement: Additional File 1 — This compressed (gz) file contains two directories tmadb_bmc_html and tmadb_bmc and two files, create_tmadb.txt and a README file which can be extracted using gunzip software. The create_tmadb.txt file contains all the MySQL create commands for creating tables contained in the database. The README file provides instructions to help the user install the software. The tmadb_bmc_html directory contains html, xml and text files required for interfacing with the cgi programs. The tmadb_bmc directory contains ten files, nine files with the extension cgi and a file named config.pl. config.pl Contains variables that require modification during installation. colo_form_input.cgi Program to upload colorectal pathology information from the Web form. colo_path_input.cgi Program to upload colorectal pathology information from the Web. core_path.cgi Program to upload specific information relating to each core from the Web. keysearch.cgi Program to query the database using a keyword search or a specific specimen identifier. mysql_search.cgi Program to query the database using MySQL statements. table_contents.cgi Program to display the contents of each table in the database. tma_construct.cgi Program to upload TMA design construct information from the Web. tma_result_input.cgi Program to upload TMA experiment protocol and results from the Web. unknown_path.cgi Program to upload pathology information from the Web for specimens where the diagnosis is unknown. [file 1471-2105-6-218-S1.gz › tmadb/tmadb_bmc_html/colo_path_input.htm]

 Submission of pathology data associated with each block specimen on TMA
  
  

Please click the browse button to select the file containg the pathology data for each of the block specimens used in the TMA for assimilation in to the database.
The file can either be an XML file or a tab delimited text file in the format specified here.
  
  
Please type in your email address:  
For colorectal cancer: 
  
  

  


  
  
To submit colorectal pathology report from a form click
Colorectal Cancer Histopathology Report.
  
For further information on the colorectal minimum data set click National minimum data set for colorectal cancer.  
  
To submit pathology for gastric cancer Gastric Cancer Histopathology Report
  
  
To submit pathology information where the type of cancer is unkown click here.
