# Supplementary material for: TmaDB: a repository for tissue microarray data
Source: BMC Bioinformatics. 2005 Sep 1;6:218. doi: 10.1186/1471-2105-6-218 (PMC1215475; doi:10.1186/1471-2105-6-218)
Supplement: Additional File 1 — This compressed (gz) file contains two directories tmadb_bmc_html and tmadb_bmc and two files, create_tmadb.txt and a README file which can be extracted using gunzip software. The create_tmadb.txt file contains all the MySQL create commands for creating tables contained in the database. The README file provides instructions to help the user install the software. The tmadb_bmc_html directory contains html, xml and text files required for interfacing with the cgi programs. The tmadb_bmc directory contains ten files, nine files with the extension cgi and a file named config.pl. config.pl Contains variables that require modification during installation. colo_form_input.cgi Program to upload colorectal pathology information from the Web form. colo_path_input.cgi Program to upload colorectal pathology information from the Web. core_path.cgi Program to upload specific information relating to each core from the Web. keysearch.cgi Program to query the database using a keyword search or a specific specimen identifier. mysql_search.cgi Program to query the database using MySQL statements. table_contents.cgi Program to display the contents of each table in the database. tma_construct.cgi Program to upload TMA design construct information from the Web. tma_result_input.cgi Program to upload TMA experiment protocol and results from the Web. unknown_path.cgi Program to upload pathology information from the Web for specimens where the diagnosis is unknown. [file 1471-2105-6-218-S1.gz › tmadb/tmadb_bmc_html/colorectalcancer.pdf]

MINIMUM DATASET FOR COLORECTAL CANCER HISTOPATHOLOGY REPORTS

Co-ordinators: Professor P. Quirke, University of Leeds
Professor G.T. Williams, University of Wales College of Medicine

These proposals for reporting of colorectal cancer should be implemented for the following reasons:

1. Patients who have lymph node involvement (Dukes stages C1 & C2, TNM stages pN1, pN2) are likely to receive adjuvant chemotherapy which is of possible benefit, mildly toxic and costly[1,2].

2. Patients with rectal adenocarcinoma and circumferential margin involvement are at high risk of local recurrence[3,4,5] and may receive post-operative radiotherapy +/- chemotherapy which is toxic and costly but may decrease the likelihood[6,7] of death from this unpleasant and nearly uniformly fatal complication. The frequency of circumferential margin involvement found may indicate the quality of rectal cancer surgery being performed[8].

3. To confirm that radical surgery was necessary and to place the patient in the appropriate stage so that the individual can be given a prognosis and surgeons can accurately audit their outcomes avoiding case mix selection bias.

4. To identify whether the anal sphincter has been lost. The frequency of abdomino-perineal resections is an indicator of the quality of surgery.

5. To allow the equitable comparison of surgeons in colorectal cancer audits [9,10] to identify good surgical practice [8] and the comparison of patients in clinical trials.

This form has been devised to include the minimum amount of data required for a careful assessment of a colorectal cancer specimen. It is evidence based and has been widely discussed. It has been approved by the Royal Colleges of Pathologists and Surgeons (England), the Associations of Coloproctology and Clinical Pathologists, The United Kingdom Coordinating Committee for Cancer Research Colorectal Cancer Subcommittee, the Scottish Intercollegiate Guidelines Network, the Welsh CROPS Project, the UK Association of Cancer Registries and the Pathology Section of the British Society of Gastroenterology. We strongly recommend its use as a minimum dataset. This document will be reviewed in 2000 and before that if new evidence emerges.

Further copies of the reporting forms are available at the end of this booklet and can also be downloaded from the College website (www.rcpath.org).

NATIONAL MINIMUM DATA SET
COLORECTAL CANCER HISTOPATHOLOGY REPORT

Surname .... Forenames .... Date of birth.......................... Sex...............

Hospital .... Hospital No .... NHS No ....

Date of receipt .... Date of reporting .... Report No............................

Pathologist .... Surgeon................................

Gross Description

Site of tumour...

Maximum tumour diameter..

Distance of tumour to nearer margin (cut end)............

Presence of tumour perforation (pT4) ☐ **Yes** ☐ **No**

For rectal tumours

Tumour is ☐ above ☐ at ☐ below
the peritoneal reflection

Distance from the dentate line ....

Histology
Type

Adenocarcinoma ☐ Yes ☐ No
(to include mucinous and signet ring adenocarcinomas)

If No, other...

Differentiation by predominant area

☐ Well / moderate ☐ Poor

Local Invasion

☐ Submucosa (pT1)

☐ Muscularis propria (pT2)

☐ Beyond muscularis propria (pT3)

☐ Tumour cells have breached the peritoneal surface or invaded adjacent organs (pT4)

Margins

**Tumour involvement**	**N/A**	**Yes**	**No**
Doughnut	☐	☐	☐
Margin (cut end)	☐	☐	☐

For rectal tumours

Metastatic Spread

No of lymph nodes examined...

No of positive lymph nodes..

(pN1 1-3 nodes, pN2 4+nodes involved)

	**Yes**	**No**
......Apical node positive (Dukes C2)	☐	☐
...Extramural vascular invasion	☐	☐

Background Abnormalities

	**Yes**	**No**
Adenoma(s)	☐	☐
_Synchronous carcinomas(s)<br>_*(Complete a separate form for each cancer)*	☐	☐
Ulcerative colitis	☐	☐
Crohn's disease	☐	☐
Familial adenomatous polyposis	☐	☐

..Other comments...
....

Pathological Staging

Complete resection at all margins ☐ **Yes** ☐ **No**

TNM

☐ T ☐ N ☐ M

Dukes

☐ Dukes A (Growth limited to wall, nodes negative)

☐ Dukes B (Growth beyond muscularis propria, nodes negative)

☐ Dukes C1 (Nodes positive and apical node negative)

Circumferential margin involvement ☐ ☐ ☐

☐ Dukes C2 (Apical node positive)

Histological measurement from tumour to circumferential margin ……..mm

Histologically confirmed liver metastases ☐ **Yes** ☐ **No**

Signature .... Date…../…../…… SNOMED Codes………/…………

NOTES ON RECORDING OF DATA ITEMS

Please record data items for all primary colorectal cancers as follows, with all measurements in mm.

GROSS DESCRIPTION

Site of Tumour

This will usually be stated on the request form. However if examination of the specimen suggests that the stated site is incorrect this should be queried with the surgeon and corrected if necessary.

Maximum tumour diameter

Measured from the luminal aspect of the bowel. The thickness of the tumour is ignored for this measurement.

Distance of tumour to nearest margin

Measured from the nearest cut end of the specimen, not the circumferential margin. It is only necessary to examine the margins histologically if tumour extends macroscopically to within 30mm of one of these. For tumours further than this it can be assumed that the cut ends are not involved. Exceptions to this recommendation are adenocarcinomas that are found on subsequent histology to have an exceptionally infiltrative growth pattern, show extensive vascular or lymphatic permeation, or are pure signet ring carcinomas, small cell carcinomas, or undifferentiated carcinomas.

Presence of tumour perforation

If the tumour has perforated into the peritoneal cavity this should be recorded. Such cases are always regarded as pT4 in the TNM staging system (see pages 8 & 9). If perforation does not involve the tumour the “No” box should be marked.

For Rectal Tumours
Relationship to the Peritoneal Reflection
The crucial landmark for recording the site of rectal tumours is the peritoneal reflection. This is identified from the exterior surface of the *anterior* aspect of the specimen (see Fig. 1).

Rectal tumours are classified according to whether they are:

a) entirely above the level of the peritoneal reflection anteriorly
b) astride (or at) the level of the peritoneal reflection anteriorly
c) entirely below the level of the peritoneal reflection anteriorly

Tumours below the peritoneal reflection have the highest rates of local recurrence.

Fig. 1

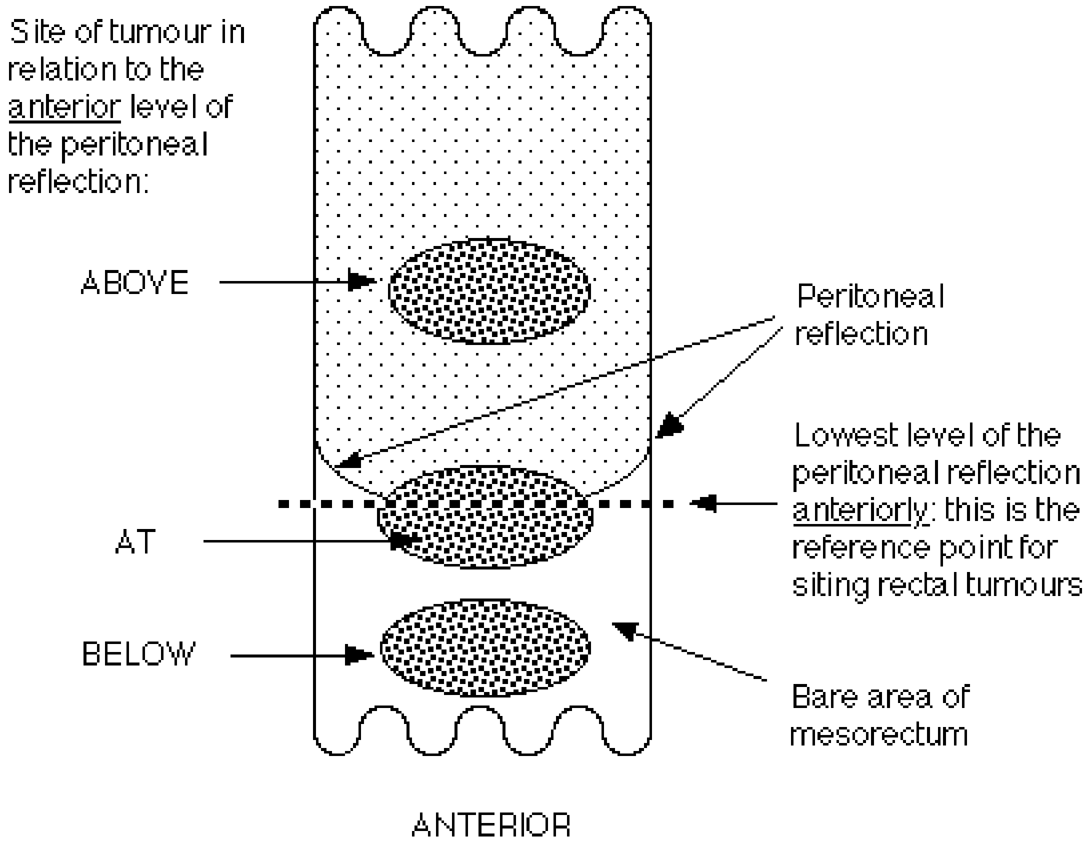

Distance from dentate line
This can only be measured for low rectal tumours in abdominoperineal excision of rectum (APER) specimens. This measurement is important to make as it identifies patients who have lost their internal sphincter.

HISTOLOGY

Type

Virtually all colorectal cancers are adenocarcinomas. Other rare forms worthy of special mention are:

- adenosquamous carcinomas
- true squamous carcinomas (not including upwardly spreading anal tumours)
- adenocarcinoid (composite carcinoma/carcinoid) tumours
- small cell carcinomas
- totally undifferentiated carcinomas

Mucinous carcinomas and signet ring carcinomas are recorded as adenocarcinomas.

Differentiation by Predominant Area

Poorly differentiated carcinomas should be separated from other types, but only if this forms the *predominant* area of the tumour. Small foci of apparent poor differentiation are not uncommon at the advancing edge of tumours, but these are insufficient to classify the tumour as poorly differentiated.

The criteria for poorly differentiated tumours are *either* irregularly folded, distorted and often small tubules *or* the absence of any tubular formation.

Local Invasion

The *maximum* degree of local invasion into or through the bowel wall is recorded, so *only one* of the four boxes should be marked.

Sufficient blocks of the tumour should be taken to assess this carefully. It is recommended that the whole tumour and attached mesentery (or mesorectum) are serially sliced at 3-4 mm intervals with a sharp knife in order to identify macroscopically the areas of deepest invasion, which should be blocked for histological confirmation.

Involvement of the serosal (peritoneal) surface is defined as the presence of tumour cells on the peritoneal surface. Thus tumour cell penetration of the serosa needs to be seen by penetration or ulceration. Note that this does *not* constitute circumferential margin involvement since there is no involvement of a retroperitoneal margin.

Margins
Tumour involvement:

Doughnuts

Strictly speaking, it is not necessary to examine doughnuts histologically if the main tumour is >30mm from the cut end of the main specimen or in other rare cases described above but this is a decision to be made locally.

When doughnuts from stapling devices are examined histologically the presence or absence of tumour is recorded. If doughnuts are not sectioned because it is deemed unnecessary locally, or if no doughnuts are submitted for examination by the surgeon, this item should be recorded as not applicable.

Margin (cut end)

When cut ends are examined histologically (see criteria above) the presence or absence of tumour should be recorded. If margins are not examined histologically they should be recorded as not applicable.

Circumferential Margin (Rectal cancers only)

Accurate assessment of the circumferential (radial) margins of these rectal tumours is very important because it influences post-operative therapy.

Note that the circumferential margin is reported only for rectal cancers; for tumours at other sites the "not applicable" box is marked. It represents involvement of the surgical margins of the connective tissues around the rectum in an area where there is no peritoneal covering, i.e. the unshaded area in Fig. 2. Hence involvement of this margin is different from, and quite unrelated to, serosal involvement.

Fig. 2

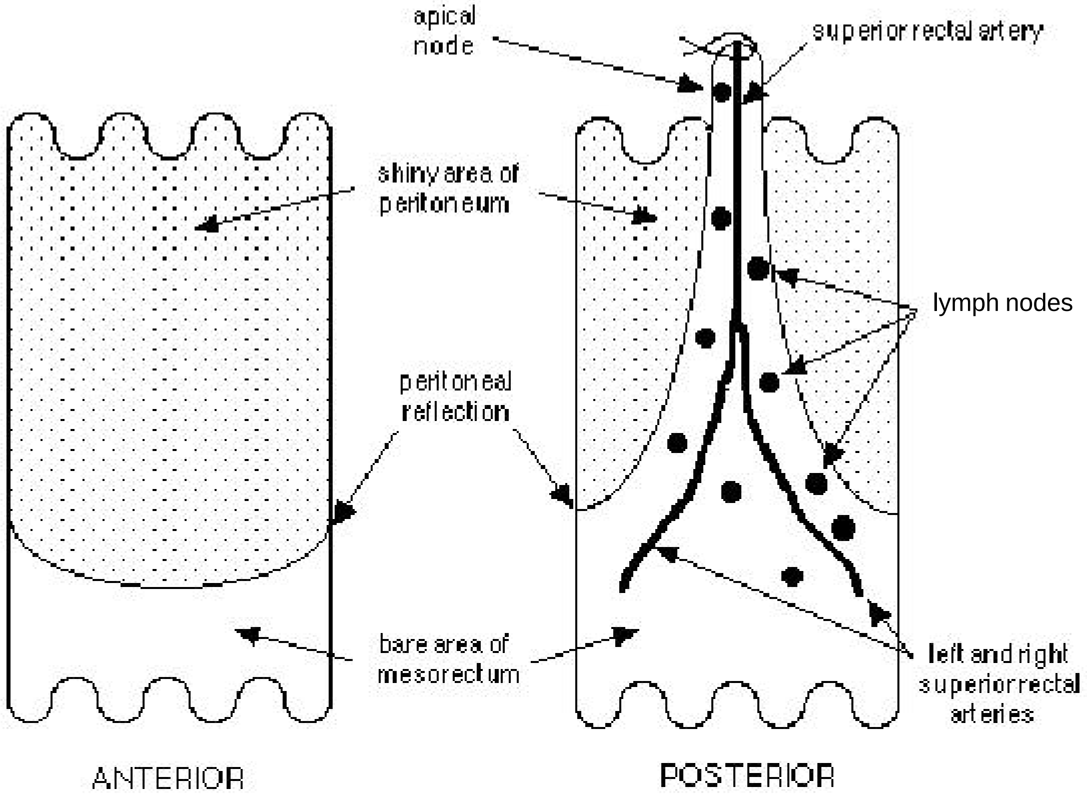

Anteriorly the rectum is covered by peritoneum and only the area below the peritoneal reflection (unshaded in Fig. 2) is at risk of circumferential margin involvement. Posteriorly this area, and the area above it, a triangular shaped bare area running up to the start of the sigmoid mesocolon, are at risk from not only direct tumour spread but also metastatic deposits in lymph nodes that lie against the circumferential margin.

It is recommended that the whole of this margin (i.e. the mesorectum) is painted with a marker such as silver nitrate or India ink before dissecting the specimen. The tumour is then best sliced serially at 3-4 mm intervals to select blocks from areas that are closest macroscopically to the circumferential margin. Slices should then be made of the area above and below the tumour to look for metastatic deposits. If lymph nodes lie against the circumferential margin then this margin should be included in the block.

The minimum distance between the tumour and the circumferential margin in millimetres is also recorded from the histological slides (see Fig. 3). If this is $\leq$1 mm then the circumferential margin is *regarded as involved* in the assessment on completeness of resection later on in the proforma. Such involvement may be through direct continuity with the main tumour, by tumour in veins, lymphatics or lymph nodes, or by tumour deposits discontinuous from the main growth.

Fig. 3

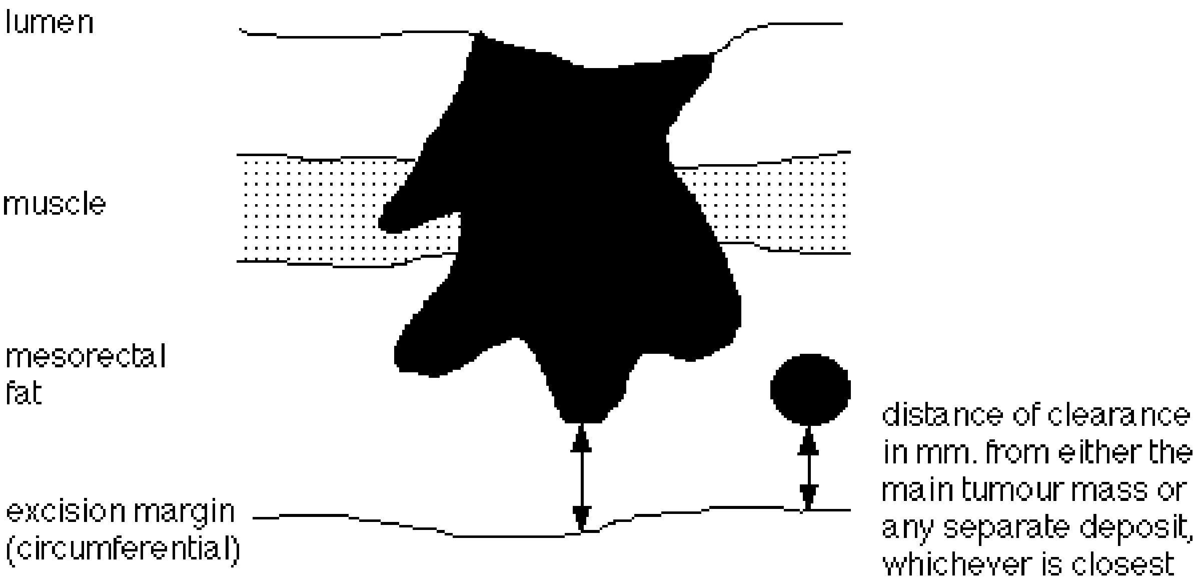

Metastatic Spread

Number of lymph nodes examined
All lymph nodes found in the specimen should be sampled and counted, regardless of their site or size.

Number of positive lymph nodes
This must be equal to or less than the number of lymph nodes sampled.

Extramural tumour deposits measuring $\geq$3 mm are counted as involved lymph nodes even if no residual lymph node structure can be identified. Smaller deposits are regarded as apparent discontinuous extensions of the main tumour.

In the TNM staging system, pN1 corresponds to involvement of 1-3 nodes and pN2 to involvement of 4 or more nodes (A previously used pN3 category was dropped in the 1997 TNM revision).

Apical node positive
For Dukes' staging the pathologist will only need to identify separately the apical lymph node closest to the main vascular tie. This is not defined by any measure of distance, but is simply the first node identified by slicing the mesentery serially and distally from the vascular tie.

Extramural vascular invasion
This is recorded when tumour is present within an extramural endothelium-lined space that is *either* surrounded by a rim of muscle *or* contains red blood cells.

Background Abnormalities

The presence or absence of the following in the background bowel is recorded:

- adenoma(s)
- synchronous carcinoma(s) (each of which will require a separate proforma)
- ulcerative colitis
- Crohn's disease
- familial adenomatous polyposis

PATHOLOGICAL STAGING

It is recommended that Dukes' and TNM staging is used. The proforma is designed for both systems.

Complete resection at all margins

This includes the doughnuts, the ends of the specimen and, for rectal tumours, the mesorectal circumferential resection plane.

Where doughnuts and the ends of the specimen are not examined histologically because the tumour is >30 mm away these are assumed to be tumour-free. Circumferential margins of rectal tumours are regarded as involved if tumour extends histologically to $\leq$1 mm from this margin.

Peritoneal (serosal) involvement alone is not reason to categorise the tumour as incompletely excised.

TNM

Here the T stage and the N stage are derived from the extent of local spread and lymph node metastases, the criteria for each stage being defined on the form. The appropriate figure is entered in each box. The pre-fix p is used to indicate pathological staging. If the patient has had preoperative chemotherapy or radiotherapy then the prefix yp should be used to indicate the stage found may not be the presenting stage of the tumour.

The following should be noted:

i In determining the pT stage, tumours that have perforated into the peritoneal cavity are regarded as pT4, irrespective of other factors.

ii Direct intramural spread of caecal carcinomas into the terminal ileum does not affect the pT stage. However direct extramural spread (across the serosa) of a colorectal carcinoma into another part of the large or small intestine corresponds to pT4.

iii Extramural deposits of tumour that are not obviously within lymph nodes are regarded as discontinuous extensions of the main tumour if they measure <3mm in diameter but as lymph nodes if they measure ≥ 3mm in diameter.

iv The difference between stage pN1 and pN2 is the number of lymph nodes involved (pN1 = 1-3 nodes, pN2 = 4+ nodes), irrespective of their site in the resection specimen.

v Pathological M staging can only be based on distant metastases that are submitted for histology by the surgeon and will therefore tend to underestimate the true M stage. Pathologists will therefore only be able to use M1 (distant metastases present) or MX (distant metastases unknown). Note that metastatic deposits in lymph nodes distant from those surrounding the main tumour or its main artery in the specimen, which will usually be submitted separately by the surgeon (e.g. in para-aortic nodes or nodes surrounding the external iliac or common iliac arteries), are counted as distant metastases and hence pM1.

Dukes

Here one of the four boxes is marked, corresponding to the Dukes' stage. Criteria used for Dukes' staging are given on the form. Note that Dukes' so-called stage D is not used.

Histologically confirmed liver metastases

Here one of the two boxes is marked. If no liver biopsy is submitted with the resection specimen then the “No” box is marked.

References

1. Moertel CG, Fleming TR, MacDonald JS, Haller DG, Laurie JA, Tangen CM et al. Fluorouracil plus levamisole as effective adjuvant therapy after resection of stage III colon carcinoma: a final report. *Ann Int Med* 1995; **122:**321-6.

2. International Multicentre Pooled Analysis of Colon Cancer Trials (IMPACT) Investigators. Efficacy of adjuvant fluorouracil and folinic acid in colon cancer. *Lancet* 1995; **345**:939-44.

3. Quirke P, Dixon MF, Durdey P, Williams NS. Local recurrence of rectal adenocarcinoma due to inadequate surgical resection: Histopathological study of lateral tumour spread and surgical excision. *Lancet* 1986; ii: 996-999.

4. Adam IJ, Mohamdee MO, Martin IG, Scott N, Finan PJ, Johnston D, Dixon MF, Quirke P. Role of circumferential margin involvement in the local recurrence of rectal cancer. *Lancet* 1994; **344:** 707-711.

5. Ng IOL, Luk ISC, Yuen ST, Lau PWK, Pritchett CJ, Ng M, Poon GP, Ho J. Surgical lateral clearance in resected rectal carcinomas. A multivariate analysis of clinicopathological features. *Cancer* 1993; **71**; 1972-76.

6. Medical Research Council Rectal Cancer Working Party. Randomised trial of surgery alone versus surgery followed by radiotherapy for mobile cancer of the rectum. *Lancet* 1996; **348**; 1610-14.

7. Thomas PR, Linbald AS. Adjuvant postoperative radiotherapy and chemotherapy in rectal carcinoma: A review of the Gastrointestinal Tumor Study Group Experience. *Radiother Oncol* 1988; **13**: 245-52.

8. Quirke P. Limitations of existing systems of staging for rectal cancer: The forgotten margin. *Rectal Cancer Surgery*. Ed. Soreide O & Norstein J. Springer-Verlag 1997, 63-81.

9. McArdle CS, Hole D. Impact of variability among surgeons on postoperative morbidity and mortality and ultimate survival. *BMJ* 1991; **302**:1501-5.

10. Hermanek P, Wiebelt H, Staimmer D, Riedi S. Prognostic factors of rectum carcinoma - experience of the German Multicentre Study SGCRC *Tumori* 1995; **81**: 60-64.

11. Cross SS, Bull AD, Smith JH. Is there any justification for the routine examination of bowel resection margins in colorectal adenocarcinoma? *J Clin Pathol* 1989; **42**(10):1040-42.

12. Halvorsen TB, Seim E. Degree of differentiation in colorectal adenocarcinomas: a multivariate analysis of the influence on survival. *J Clin Pathol* 1988; **41**(5):532-537.

13. Shepherd NA, Baxter KJ, Love SB. The prognostic importance of peritoneal involvement in colonic cancer: a prospective evaluation. *Gastroenterol* 1997;**112**(4):1096-1102**.**
