# Supplementary material for: TmaDB: a repository for tissue microarray data
Source: BMC Bioinformatics. 2005 Sep 1;6:218. doi: 10.1186/1471-2105-6-218 (PMC1215475; doi:10.1186/1471-2105-6-218)
Supplement: Additional File 1 — This compressed (gz) file contains two directories tmadb_bmc_html and tmadb_bmc and two files, create_tmadb.txt and a README file which can be extracted using gunzip software. The create_tmadb.txt file contains all the MySQL create commands for creating tables contained in the database. The README file provides instructions to help the user install the software. The tmadb_bmc_html directory contains html, xml and text files required for interfacing with the cgi programs. The tmadb_bmc directory contains ten files, nine files with the extension cgi and a file named config.pl. config.pl Contains variables that require modification during installation. colo_form_input.cgi Program to upload colorectal pathology information from the Web form. colo_path_input.cgi Program to upload colorectal pathology information from the Web. core_path.cgi Program to upload specific information relating to each core from the Web. keysearch.cgi Program to query the database using a keyword search or a specific specimen identifier. mysql_search.cgi Program to query the database using MySQL statements. table_contents.cgi Program to display the contents of each table in the database. tma_construct.cgi Program to upload TMA design construct information from the Web. tma_result_input.cgi Program to upload TMA experiment protocol and results from the Web. unknown_path.cgi Program to upload pathology information from the Web for specimens where the diagnosis is unknown. [file 1471-2105-6-218-S1.gz › tmadb/tmadb_bmc_html/core_path_input.htm]

 TMA experimental protocol and result submission page
  
  

Please click on the browse button to select the file containing the pathological data for each of the core on a particular TMA block for assimilation into the database.
The file can either be an XML file or a tab delimited text file in the format specified below.
  
  
Please type in your email address:
  

  
  

  

  
  
Please click on the column headings for a detailed explanation, these are adapted from the  Tissue MicroArray Common Data Elements devloped by the API community.
  
  
TMA core details format:
This is an excel tab delimited text file, see template file.  
  
  

|  |  |  |  |  |  |  |  |
| --- | --- | --- | --- | --- | --- | --- | --- |
| Specimen\_id | Core\_organism | Tissue\_origin | Donor\_block\_drill\_site | Drill\_site\_diagnosis | Anatomic\_site | Quality\_of\_core | Comments |
| N-855/99 | - | - | - | - | - | - | - |
| T-855/99 | - | - | - | - | - | - | - |
| N-853/99 | - | - | - | - | - | - | - |
| T-853/99 | - | - | - | - | - | - | - |
| N-852/99 | - | - | - | - | - | - | - |
| T-852/99 | - | - | - | - | - | - | - |
| N-192/00 | - | - | - | - | - | - | - |
| T-192/00 | - | - | - | - | - | - | - |
| N-1011/99 | - | - | - | - | - | - | - |
| T-1011/99 | - | - | - | - | - | - | - |
| N-989/99 | - | - | - | - | - | - | - |
| T-989/99 | - | - | - | - | - | - | - |
| N-988/99 | - | - | - | - | - | - | - |
| T-988/99 | - | - | - | - | - | - | - |
| N-683/97 | - | - | - | - | - | - | - |
| T-683/97 | - | - | - | - | - | - | - |
| N-681/97 | - | - | - | - | - | - | - |
| T-681/97 | - | - | - | - | - | - | - |
| N-652/97 | - | - | - | - | - | - | - |
| T-1011/99 | - | - | - | - | - | - | - |
| N-200/00 | - | - | - | - | - | - | - |
| T-200/00 | - | - | - | - | - | - | - |
| N-199/00 | - | - | - | - | - | - | - |
| T-199/00 | - | - | - | - | - | - | - |
| N-838/97 | - | - | - | - | - | - | - |
| T-838/97 | - | - | - | - | - | - | - |
| N-748/97 | - | - | - | - | - | - | - |
| T-748/97 | - | - | - | - | - | - | - | |
| N-720/97 | - | - | - | - | - | - | - |
| T-720/97 | - | - | - | - | - | - | - |
| N-719/97 | - | - | - | - | - | - | - |
| T-719/97 | - | - | - | - | - | - | - |
| N-718/97 | - | - | - | - | - | - | - |
| T-718/97 | - | - | - | - | - | - | - |
| N-717/97 | - | - | - | - | - | - | - |
| T-717/97 | - | - | - | - | - | - | - |
| N-909/97 | - | - | - | - | - | - | - |
| T-909/97 | - | - | - | - | - | - | - |
| N-908/97 | - | - | - | - | - | - | - |
| T-908/97 | - | - | - | - | - | - | - |
| N-906/97 | - | - | - | - | - | - | - |
| T-906/97 | - | - | - | - | - | - | - |
| N-905/97 | - | - | - | - | - | - | - |
| T-905/97 | - | - | - | - | - | - | - |
| N-642/98 | - | - | - | - | - | - | - |
| T-642/98 | - | - | - | - | - | - | - |
| N-641/98 | - | - | - | - | - | - | - |
| T-641/98 | - | - | - | - | - | - | - |
| N-640/98 | - | - | - | - | - | - | - |
| T-640/98 | - | - | - | - | - | - | - |
| N-639/98 | - | - | - | - | - | - | - |
| T-639/98 | - | - | - | - | - | - | - |
| N-1060/97 | - | - | - | - | - | - | - |
| T-1060/97 | - | - | - | - | - | - | - |
| N-1059/97 | - | - | - | - | - | - | - |
| T-1059/97 | - | - | - | - | - | - | - |
| N-1058/97 | - | - | - | - | - | - | - | - |
| T-1058/97 | - | - | - | - | - | - | - | - |
| N-1057/97 | - | - | - | - | - | - | - | - |
| T-1057/97 | - | - | - | - | - | - | - |
| N-196/00 | - | - | - | - | - | - | - |
| T-196/00 | - | - | - | - | - | - | - |
| N-195/00 | - | - | - | - | - | - | - |
| T-195/00 | - | - | - | - | - | - | - |
| N-194/00 | - | - | - | - | - | - | - |
| T-194/00 | - | - | - | - | - | - | - |
| N-934/99 | - | - | - | - | - | - | - |
| T-934/99 | - | - | - | - | - | - | - |
| N-930/99 | - | - | - | - | - | - | - |
| T-930/99 | - | - | - | - | - | - | - |
| N-715/97 | - | - | - | - | - | - | - |
| T-715/97 | - | - | - | - | - | - | - |
| N-684/97 | - | - | - | - | - | - | - |
| T-684/97 | - | - | - | - | - | - | - |
| N-1428/98 | - | - | - | - | - | - | - |
| T-1428/98 | - | - | - | - | - | - | - |
| N-1287/98 | - | - | - | - | - | - | - |
| T-1287/98 | - | - | - | - | - | - | - |
| N-1176/98 | - | - | - | - | - | - | - |
| T-1176/98 | - | - | - | - | - | - | - |

  
  
Specimen\_id 
This is the block specimen identification number.
  
  
Core\_organism 
Datatype: Character String representing taxonomy.dat indentifier number followed by
an allowable taxonomy.dat name for the identifier number  
Maximum Occurrence: Unlimited  
Definition: Organism name at species level for organism whose tissue is represented in the donor block,  
Comment: URI for taxonomy.dat is ftp://ftp.ebi.ac.uk/pub/databases/taxonomy/taxonomy.dat  
The correct entry for human tissue is "9606 human"
  
  
Tissue\_origin 
  
  
  Donor\_block\_drill\_site   
Datatype: Character String   
Maximum Occurrence: Unlimited   
Definition: This is the location in the donor block from which
the core was taken.  
Comment: It can be provided as coordinates or as description, but
the description of the site must be explained adequately
within this document.
  
  
 Drill\_site\_diagnosis   
Datatype: Character String
  
Definition: This is the diagnosis for the specific tissue region
for the core as drilled from the donor. For example, this might
be lung adenocarcinoma or histopathologically normal or emphysema.
This is free text. However, in the interest of facilitating searches
of the database, we would urge users to adhere to NCI accepted
nomenclature where defined.
  
  
  Anatomic\_site 
Datatype: Character String   
Maximum Occurrence: Unlimited   
Definition: Synonymous with topography, a text string-would like to introduce a controlled
vocabulary or ontology here. We will use a controlled vocabulary from UMLS
Comment:
  
  
Quality\_of\_core  
Datatype: Character String  
Maximum Occurrence: Unlimited  
Definition: Comment specifically as Adequate or Not Adequate for quality of core to represent
donor block diagnosis info specified for this core. Indicate if core has fallen off.
  
  
 Comment    
Datatype: Character String  
Maximum Occurrence: Unlimited  
Definition: Comment specifically as Adequate or Not Adequate for quality of core to represent
donor block diagnosis info specified for this core. Indicate if core has fallen off.
  
  
