# Supplementary material for: TmaDB: a repository for tissue microarray data
Source: BMC Bioinformatics. 2005 Sep 1;6:218. doi: 10.1186/1471-2105-6-218 (PMC1215475; doi:10.1186/1471-2105-6-218)
Supplement: Additional File 1 — This compressed (gz) file contains two directories tmadb_bmc_html and tmadb_bmc and two files, create_tmadb.txt and a README file which can be extracted using gunzip software. The create_tmadb.txt file contains all the MySQL create commands for creating tables contained in the database. The README file provides instructions to help the user install the software. The tmadb_bmc_html directory contains html, xml and text files required for interfacing with the cgi programs. The tmadb_bmc directory contains ten files, nine files with the extension cgi and a file named config.pl. config.pl Contains variables that require modification during installation. colo_form_input.cgi Program to upload colorectal pathology information from the Web form. colo_path_input.cgi Program to upload colorectal pathology information from the Web. core_path.cgi Program to upload specific information relating to each core from the Web. keysearch.cgi Program to query the database using a keyword search or a specific specimen identifier. mysql_search.cgi Program to query the database using MySQL statements. table_contents.cgi Program to display the contents of each table in the database. tma_construct.cgi Program to upload TMA design construct information from the Web. tma_result_input.cgi Program to upload TMA experiment protocol and results from the Web. unknown_path.cgi Program to upload pathology information from the Web for specimens where the diagnosis is unknown. [file 1471-2105-6-218-S1.gz › tmadb/tmadb_bmc_html/help.htm]

# TmaDB

The homepage describes the tissue microarray technique and its advantages.
The menus on the left margin:- 1. Search TmaDB
  Allows users to query the database for specific keywords in each of the tables within the database.
  2. Display all records
  Displays the ER (entity relation) diagram illustrating all the tables and their attributes.
  3. Display all records
  Allows users to view all the data that is stored in each of the tables within the database. It is display as a table containing all the table names within the database as a link, which upon clicking displays the entire content of that table.
  4. MySQL search
  This page is for the benefit of users who are familiar with MySQL command to enable direct examination of the database. However the user does not have privileges to update and insert data from this page.
  5. Submit TMA design
  This page allows users to submit the TMA construction details to be assimilated into the database. The data must be in format specified on the page.
  6. Submit core path data
  Submission of data relating specifically to each of the cores on the tma slides.
  7. Submit experimental protocol and results
  Submission of staining protocol and the staining results for each of the cores.
  8. Submit block path report
  Submission of pathology reports for each of the cores on the TMA slide.
  If there are any queries relating to the database regarding submission of data or querying the database please email  Archana Sharma-Oates
