# Supplementary material for: TmaDB: a repository for tissue microarray data
Source: BMC Bioinformatics. 2005 Sep 1;6:218. doi: 10.1186/1471-2105-6-218 (PMC1215475; doi:10.1186/1471-2105-6-218)
Supplement: Additional File 1 — This compressed (gz) file contains two directories tmadb_bmc_html and tmadb_bmc and two files, create_tmadb.txt and a README file which can be extracted using gunzip software. The create_tmadb.txt file contains all the MySQL create commands for creating tables contained in the database. The README file provides instructions to help the user install the software. The tmadb_bmc_html directory contains html, xml and text files required for interfacing with the cgi programs. The tmadb_bmc directory contains ten files, nine files with the extension cgi and a file named config.pl. config.pl Contains variables that require modification during installation. colo_form_input.cgi Program to upload colorectal pathology information from the Web form. colo_path_input.cgi Program to upload colorectal pathology information from the Web. core_path.cgi Program to upload specific information relating to each core from the Web. keysearch.cgi Program to query the database using a keyword search or a specific specimen identifier. mysql_search.cgi Program to query the database using MySQL statements. table_contents.cgi Program to display the contents of each table in the database. tma_construct.cgi Program to upload TMA design construct information from the Web. tma_result_input.cgi Program to upload TMA experiment protocol and results from the Web. unknown_path.cgi Program to upload pathology information from the Web for specimens where the diagnosis is unknown. [file 1471-2105-6-218-S1.gz › tmadb/tmadb_bmc_html/keysearch_db.htm]

 Magic Targets: main


  

This pages allows a keyword search of the entire database or the individual tables within the database.  
  
 
Click here to view table attributes.
  
  

To search the entire database select database from the drop down menu below and type in a keyword and then click on the submit button.
  
  
Else to search the individual tables select the table you wish to search and enter a keyword in the textbox and then click on the submit button.
  
  
Search:
database
patient
path\_report
block
tma
tma\_experiment
core\_info
core\_expt\_result
whole\_tissue\_section
for keyword
Robinson


  

  
  
  
  
