# Supplementary material for: TmaDB: a repository for tissue microarray data
Source: BMC Bioinformatics. 2005 Sep 1;6:218. doi: 10.1186/1471-2105-6-218 (PMC1215475; doi:10.1186/1471-2105-6-218)
Supplement: Additional File 1 — This compressed (gz) file contains two directories tmadb_bmc_html and tmadb_bmc and two files, create_tmadb.txt and a README file which can be extracted using gunzip software. The create_tmadb.txt file contains all the MySQL create commands for creating tables contained in the database. The README file provides instructions to help the user install the software. The tmadb_bmc_html directory contains html, xml and text files required for interfacing with the cgi programs. The tmadb_bmc directory contains ten files, nine files with the extension cgi and a file named config.pl. config.pl Contains variables that require modification during installation. colo_form_input.cgi Program to upload colorectal pathology information from the Web form. colo_path_input.cgi Program to upload colorectal pathology information from the Web. core_path.cgi Program to upload specific information relating to each core from the Web. keysearch.cgi Program to query the database using a keyword search or a specific specimen identifier. mysql_search.cgi Program to query the database using MySQL statements. table_contents.cgi Program to display the contents of each table in the database. tma_construct.cgi Program to upload TMA design construct information from the Web. tma_result_input.cgi Program to upload TMA experiment protocol and results from the Web. unknown_path.cgi Program to upload pathology information from the Web for specimens where the diagnosis is unknown. [file 1471-2105-6-218-S1.gz › tmadb/tmadb_bmc_html/main.htm]

 Magic Targets: main


  

**What is tissue microarray (TMA)?**
  
  

Tissue microarray (TMA) is a high-throughput method of analysing a number of potential protein targets on a large cohort of tissue specimens.
This technique enables the analysis of protein and mRNA distribution at a cellular level (in the tissue context) of thousands of tissue specimens in parallel.
The TMA technology was developed to enable genome-scale molecular pathology studies (Kononen et al., 1998).
With conventional methods (whole tissue sections) only three hundred 5 mm sections can be cut from an average sized clinical tissue specimen for use in molecular analyses (such as H&E staining, DNA fluorescence *in situ* hybridisation (FISH), mRNA *in situ* hybridisation (ISH) and immunostaining).
This therefore restricts the number genes to be analysed to 300 from the predicted ~35,000 genes in the human genome. TMAs have the advantage of assaying all the predicted genes in the human genome in parallel as a single experiment.
  
  

**How are TMAs constructed?**
  

TMAs are constructed by punching cylindrical cores of diameter 0.6 mm from tumour/normal areas of a particular specimen termed "donor" tissue block and placed into a recipient paraffin block using a custom made precision instrument.
The number of spots on a single slide is variable depending on the array design, the current maximum with the 0.6 mm needle is about 600-800 spots per standard glass microscope slide.
  

**Advantages of TMAs**
  
There are numerous advantages to this technology including:  

Up to 10,000 fold amplification of limited tissue resource.

Experimental uniformity (TMAs allow the entire cohort to be analyzed in one batch on a single slide thus reagent concentrations are identical for each case, as are incubation times and temperatures, wash conditions etc.).

Decreased assay volume (only a very small (a few ml) amount of reagent is required to analyze an entire cohort).

Does not destroy original block for diagnosis (the block may be cored a few times).

**Disadvantages of TMAs**
  
Tissue heterogeneity, a single tissue core may not be representative of the overall state of the organ.   

Tissue loss during sectioning and staining affects ~10-30% of specimens (Hoos and Cordon-Cardo, 2001).   

Orientation of array is crucial as confusion can arise. (To overcome this specimens with distinct morphology are placed outside the geometric margins of the array.)   

**References**

Hoos A, Cordon-Cardo C. (2001) Tissue microarray profiling of cancer specimens and cell lines: opportunities and limitations. Lab Invest. 81(10):1331-8.
  
  
Kononen J, Bubendorf L, Kallioniemi A, Barlund M, Schraml P, Leighton S, Torhorst J, Mihatsch MJ, Sauter G, Kallioniemi OP. (1998) Tissue microarrays for high-throughput molecular profiling of tumor specimens.Nat Med. 4(7):844-7.

  
  
This database is developed and maintained by Archana Sharma-Oates.
