# Supplementary material for: TmaDB: a repository for tissue microarray data
Source: BMC Bioinformatics. 2005 Sep 1;6:218. doi: 10.1186/1471-2105-6-218 (PMC1215475; doi:10.1186/1471-2105-6-218)
Supplement: Additional File 1 — This compressed (gz) file contains two directories tmadb_bmc_html and tmadb_bmc and two files, create_tmadb.txt and a README file which can be extracted using gunzip software. The create_tmadb.txt file contains all the MySQL create commands for creating tables contained in the database. The README file provides instructions to help the user install the software. The tmadb_bmc_html directory contains html, xml and text files required for interfacing with the cgi programs. The tmadb_bmc directory contains ten files, nine files with the extension cgi and a file named config.pl. config.pl Contains variables that require modification during installation. colo_form_input.cgi Program to upload colorectal pathology information from the Web form. colo_path_input.cgi Program to upload colorectal pathology information from the Web. core_path.cgi Program to upload specific information relating to each core from the Web. keysearch.cgi Program to query the database using a keyword search or a specific specimen identifier. mysql_search.cgi Program to query the database using MySQL statements. table_contents.cgi Program to display the contents of each table in the database. tma_construct.cgi Program to upload TMA design construct information from the Web. tma_result_input.cgi Program to upload TMA experiment protocol and results from the Web. unknown_path.cgi Program to upload pathology information from the Web for specimens where the diagnosis is unknown. [file 1471-2105-6-218-S1.gz › tmadb/tmadb_bmc_html/tma_construct.htm]

 TMA design submission page
  
  

Altogether there are four files that should be submitted to the database. They don't all need to be submitted at the same time (although they can be) however they do need to be submitted sequentially.
  
  
**1.** Please click the browse button to select the file containg the TMA construct data for assimilation in to the database.
The file can either be an XML file or a tab delimited text file in the format specified here.
  
  

Please enter your name and email address:  
Name:
  
Email:
  
  

  
  

  

  
  
 **2.**  To submit details regarding each of the cores on the particular TMA please click here.  
  
**3.**To input experimental protocol and results data please click here.  
  
**4.**  To submit clincopathological data please click  here.  
  
 
    
  
TMA design construct format: This is a tab delimited text file, see template.  
  

|  |  |  |  |  |  |
| --- | --- | --- | --- | --- | --- |
| TMA\_id=AY | Date=09/02 | Name\_of creator=Dave Robinson | Number of cores=77,7,11 | Core\_size=0.6mm | Core\_spacing=3.5mm,1.5mm |

|  |
| --- |
| TMA\_construction\_comments=none |
| TMA\_block\_storage= freezer x, shelf y | TMA\_status=exhausted | Date\_last\_updated=yymmdd | Name\_of\_person=lastname,firstname,middle initials |

|  |  |  |  |  |  |  |
| --- | --- | --- | --- | --- | --- | --- |
| N-855/99 | T-855/99 | N-853/99 | T-853/99 | N-852/99 | T-852/99 | BLANK |
| N-192/00 | T-192/00 | N-1011/99 | T-1011/99 | N-989/99 | T-989/99 | N-988/99 |
| N-683/97 | T-683/97 | N-681/97 | T-681/97 | N-293/97 | T-293/97 | N-652/97 |
| N-200/00 | T-200/00 | N-199/00 | T-199/00 | N-838/97 | T-838/97 | N-748/97 |
| N-720/97 | T-720/97 | N-719/97 | T-719/97 | N-718/97 | T-718/97 | N-717/97 |
| N-909/97 | T-909/97 | N-908/97 | T-908/97 | N-906/97 | T-906/97 | N-905/97 |
| BLANK | T-642/98 | N-641/98 | T-641/98 | N-640/98 | T-640/98 | N-639/98 |
| BLANK | T-1060/97 | BLANK | T-1059/97 | N-1058/97 | T-1058/97 | N-1057/97 |
| N-196/00 | T-196/00 | N-195/00 | T-195/00 | N-194/00 | T-194/99 | BLANK |
| N-934/99 | T-934/99 | N-930/99 | T-930/99 | N-715/97 | T-715/97 | N-684/97 |
| N-1428/98 | T-1428/98 | N-1287/98 | T-1287/98 | N-1176/98 | T-1176/98 | BLANK |

  
 KEY:  
TMA\_id can be alphanumeric (user assigned id).  
Date: date when the TMA was constructed (YYYY/MM/DD)  
Name\_of creator: Name of person who made the TMA construct.  
Number of cores: The first number "77" refers to the number of cores, the second number separated by a comma is the number of columns and finally the last number separated by a comma is the number of rows.  
Core\_size: diameter of the core needle used in the construction of TMA.  
Core\_spacing: distance between cores, the first number indicates the width followed by the height (mm).   
TMA\_construction\_comments: text description of any observation can be recorded, this field is of variable length.  
TMA\_block\_storage: description of the location where the TMA block is stored.  
  
TMA\_status: Current status if known of the TMA block; it can have one of three values: Production, Retired, Exhausted, or Unknown.  
Date\_last\_updated The date the TMA\_status was last updated.   
Name\_of\_person Name od person who last updated the TMA\_staus.  
"N-855/99" is the block\_specimen\_id of each core, where "N" refers to "normal" and "T" refers to tumour. Either N or T must be followed by - which is then followed by the block\_specimen\_id. There should no spaces in between.  
BLANK denotes cells where there are no cores present due to various reasons (may have fallen off during construction).  
  
Please Note: DO NOT LEAVE ANY CELLS EMPTY within the TMA grid.   
