# Supplementary material for: TmaDB: a repository for tissue microarray data
Source: BMC Bioinformatics. 2005 Sep 1;6:218. doi: 10.1186/1471-2105-6-218 (PMC1215475; doi:10.1186/1471-2105-6-218)
Supplement: Additional File 1 — This compressed (gz) file contains two directories tmadb_bmc_html and tmadb_bmc and two files, create_tmadb.txt and a README file which can be extracted using gunzip software. The create_tmadb.txt file contains all the MySQL create commands for creating tables contained in the database. The README file provides instructions to help the user install the software. The tmadb_bmc_html directory contains html, xml and text files required for interfacing with the cgi programs. The tmadb_bmc directory contains ten files, nine files with the extension cgi and a file named config.pl. config.pl Contains variables that require modification during installation. colo_form_input.cgi Program to upload colorectal pathology information from the Web form. colo_path_input.cgi Program to upload colorectal pathology information from the Web. core_path.cgi Program to upload specific information relating to each core from the Web. keysearch.cgi Program to query the database using a keyword search or a specific specimen identifier. mysql_search.cgi Program to query the database using MySQL statements. table_contents.cgi Program to display the contents of each table in the database. tma_construct.cgi Program to upload TMA design construct information from the Web. tma_result_input.cgi Program to upload TMA experiment protocol and results from the Web. unknown_path.cgi Program to upload pathology information from the Web for specimens where the diagnosis is unknown. [file 1471-2105-6-218-S1.gz › tmadb/tmadb_bmc_html/tma_result_input.htm]

 TMA experimental protocol and result submission page
  
  

Please click on the browse button to select the file containg the TMA expeimental and result data for assimilation into the database.
The file can either be an XML file or a tab delimited text file in the format specified here.
  
  
Please type in your email address:   

  
  

  

  
  
Please click here to view the Tissue MicroArray Common Data Elements which provides explanations for each of the headings (indicated in red).
  
  


|This is an excel tab delimited text file.  
TMA experimental protocol and results format: This is an excel tab delimited text filer, see template.txt.  

|  |
  
| Experiment\_title=SMAD4 staining | TMA\_id=AY |

|  |
| --- |
| tma\_image\_filename=colo1.jpg |

|  |  |
| --- | --- |
| Slide\_cut\_date=2003-10-02 | Section\_thickness=0.3mm |

|  |
| --- |
| Slide\_sectioning\_protocol=unkown |

|  |  |  |  |
| --- | --- | --- | --- |
| Name\_of\_antibody/stain=SMAD4 | Antibody/stain\_manufacturers\_details=Sigma | Date\_of\_experiment=2003-12-01 | Name\_of\_experimentor=Gater Helen L |

|  |
| --- |
| Experimental\_protocol=helen's protocol |
| Comments=none |

|  |  |  |  |  |  |  |  |  |  |  |  |  |  |  |  |
| --- | --- | --- | --- | --- | --- | --- | --- | --- | --- | --- | --- | --- | --- | --- | --- |
| Specimen\_id | Method\_of\_reading\_data | Names\_of\_individuals\_reading\_results | Notes | No\_of\_cells\_counted | No\_of\_nuclei\_counted | Intensity\_of\_staining | %tissue\_staining | Compartment\_staining | Staining\_pattern | Core\_result | Other\_comments | Core\_image\_filename | Image\_format | Image\_magnification | Other\_comments\_on\_images |
| N-855/99 | - | Helen | - | - | - | nc++ | - | Nc | - | Clearly positive | - | - | - | - | - |
| T-855/99 | - | Helen | - | - | - | nc+ | - | Nc | - | slightly positive | - | - | - | - | - |
| N-853/99 | - | Helen | - | - | - | nc++ | - | Nc | - | Clearly positive | - | - | - | - | - |
| T-853/99 | - | Helen | - | - | - | nc+ | - | Nc | - | slightly positive | - | - | - | - | - |
| N-852/99 | - | Helen | - | - | - | n+ | - | N | - | slightly positive | - | - | - | - | - |
| T-852/99 | - | Helen | - | - | - | nc+ | - | Nc | - | slightly positive | - | - | - | - | - |
| N-192/00 | - | Helen | - | - | - | n++ | - | N | - | Clearly positive | - | - | - | - | - |
| T-192/00 | - | Helen | - | - | - | nc+ | - | Nc | - | slightly positive | - | - | - | - | - |
| N-1011/99 | - | Helen | - | - | - | n++ | - | N | - | Clearly positive | - | - | - | - | - |
| T-1011/99 | - | Helen | - | - | - | - | - | - | - | Missing | - | - | - | - | - |
| N-989/99 | - | Helen | - | - | - | nc++ | - | Nc | - | Clearly positive | - | - | - | - | - |
| T-989/99 | - | Helen | - | - | - | - | - | - | - | Missing | - | - | - | - | - |
| N-988/99 | - | Helen | - | - | - | - | - | - | - | Missing | - | - | - | - | - |
| T-988/99 | - | Helen | - | - | - | neg | - | neg | - | Negative | - | - | - | - | - |
| N-683/97 | - | Helen | - | - | - | nc++ | - | Nc | - | Clearly positive | - | - | - | - | - |
| T-683/97 | - | Helen | - | - | - | neg | - | neg | - | Negative | - | - | - | - | - |
| N-681/97 | - | Helen | - | - | - | - | - | - | - | Missing | - | - | - | - | - |
| T-681/97 | - | Helen | - | - | - | n+ | - | N | - | slightly positive | - | - | - | - | - |
| N-652/97 | - | Helen | - | - | - | n++ | - | N | - | Clearly positive | - | - | - | - | - |
| T-1011/99 - | Helen | - | - | - | n+ | - | N | - | slightly positive | - | - | - | - | - |
| N-200/00 | - | Helen | - | - | - | nc++ | - | Nc | - | Clearly positive | - | - | - | - | - |
| T-200/00 | - | Helen | - | - | - | nc+ | - | Nc | - | slightly positive | - | - | - | - | - |
| N-199/00 | - | Helen | - | - | - | - | - | - | - | Missing | - | - | - | - | - |
| T-199/00 | - | Helen | - | - | - | nc+ | - | Nc | - | slightly positive | - | - | - | - | - |
| N-838/97 | - | Helen | - | - | - | - | - | - | - | Missing | - | - | - | - | - |
| T-838/97 | - | Helen | - | - | - | n+ | - | N | - | slightly positive | - | - | - | - | - |
| N-748/97 | - | Helen | - | - | - | - | - | - | - | Missing | - | - | - | - | - |
| T-748/97 | - | Helen | - | - | - | n+ | - | N | - | slightly positive | - | - | - | - | - |
| N-720/97 | - | Helen | - | - | - | - | - | - | - | Missing | - | - | - | - | - |
| T-720/97 | - | Helen | - | - | - | nc+ | - | Nc | - | slightly positive | - | - | - | - | - |
| N-719/97 | - | Helen | - | - | - | c+ | - | C | - | slightly positive | - | - | - | - | - |
| T-719/97 | - | Helen | - | - | - | nc+ | - | Nc | - | slightly positive | - | - | - | - | - |
| N-718/97 | - | Helen | - | - | - | - | - | - | - | Missing | - | - | - | - | - |
| T-718/97 | - | Helen | - | - | - | nc+ | - | Nc | - | slightly positive | - | - | - | - | - |
| N-717/97 | - | Helen | - | - | - | - | - | - | - | Missing | - | - | - | - | - |
| T-717/97 | - | Helen | - | - | - | nc+ | - | Nc | - | slightly positive | - | - | - | - | - |
| N-909/97 | - | Helen | - | - | - | - | - | - | - | Missing | - | - | - | - | - |
| T-909/97 | - | Helen | - | - | - | neg | - | neg | - | Missing | - | - | - | - | - |
| N-908/97 | - | Helen | - | - | - | nc++ | - | Nc | - | Clearly positive | - | - | - | - | - |
| T-908/97 | - | Helen | - | - | - | neg | - | neg | - | Missing | - | - | - | - | - |
| N-906/97 | - | Helen | - | - | - | nc++ | - | Nc | - | Clearly positive | - | - | - | - | - |
| T-906/97 | - | Helen | - | - | - | nc+ | - | Nc | - | slightly positive | - | - | - | - | - |
| N-905/97 | - | Helen | - | - | - | - | - | - | - | Missing | - | - | - | - | - |
| T-905/97 | - | Helen | - | - | - | nc+ | - | Nc | - | slightly positive | - | - | - | - | - |
| N-642/98 | - | Helen | - | - | - | - | - | - | - | Missing | - | - | - | - | - |
| T-642/98 | - | Helen | - | - | - | n+ | - | N | - | slightly positive | - | - | - | - | - |
| N-641/98 | - | Helen | - | - | - | nc++ | - | Nc | - | Clearly positive | - | - | - | - | - |
| T-641/98 | - | Helen | - | - | - | n+ | - | N | - | slightly positive | - | - | - | - | - |
| N-640/98 | - | Helen | - | - | - | nc++ | - | Nc | - | Clearly positive | - | - | - | - | - |
| T-640/98 | - | Helen | - | - | - | - | - | - | - | Missing | - | - | - | - | - |
| N-639/98 | - | Helen | - | - | - | nc++ | - | Nc | - | Clearly positive | - | - | - | - | - |
| T-639/98 | - | Helen | - | - | - | - | - | - | - | Missing | - | - | - | - | - |
| N-1060/97 | - | Helen | - | - | - | - | - | - | - | Missing | - | - | - | - | - |
| T-1060/97 | - | Helen | - | - | - | n+ | - | N | - | slightly positive | - | - | - | - | - |
| N-1059/97 | - | Helen | - | - | - | - | - | - | - | Missing | - | - | - | - | - |
| T-1059/97 | - | Helen | - | - | - | n+ | - | N | - | slightly positive | - | - | - | - | - |
| N-1058/97 | - | Helen | - | - | - | - | - | - | - | Missing | - | - | - | - | - |
| T-1058/97 | - | Helen | - | - | - | neg | - | neg | - | Negative | - | - | - | - | - |
| N-1057/97 | - | Helen | - | - | - | - | - | - | - | Missing | - | - | - | - | - |
| T-1057/97 | - | Helen | - | - | - | neg | - | neg | - | Negative | - | - | - | - | - |
| N-196/00 | - | Helen | - | - | - | nc+ | - | Nc | - | slightly positive | - | - | - | - | - |
| T-196/00 | - | Helen | - | - | - | - | - | - | - | Missing | - | - | - | - | - |
| N-195/00 | - | Helen | - | - | - | nc+ | - | Nc | - | slightly positive | - | - | - | - | - |
| T-195/00 | - | Helen | - | - | - | - | - | - | - | Missing | - | - | - | - | - |
| N-194/00 | - | Helen - | - | - | nc++ | - | Nc | - | Clearly positive | - | - | - | - | - |
| T-194/00 | - | Helen | - | - | - | - | - | - | - | Missing | - | - | - | - | - |
| N-934/99 | - | Helen | - | - | - | neg | - | None | - | Negative | - | - | - | - | - |
| T-934/99 | - | Helen | - | - | - | - | - | - | - | Missing | - | - | - | - | - |
| N-930/99 | - | Helen | - | - | - | c+ | - | C | - | slightly positive | - | - | - | - | - |
| T-930/99 | - | Helen | - | - | - | nc+ | - | Nc | - | slightly positive | - | - | - | - | - |
| N-715/97 | - | Helen | - | - | - | nc+ | - | Nc | - | slightly positive | - | - | - | - | - |
| T-715/97 | - | Helen | - | - | - | nc+ | - | Nc | - | slightly positive | - | - | - | - | - |
| N-684/97 | - | Helen | - | - | - | n+ | - | N | - | slightly positive | - | - | - | - | - |
| T-684/97 | - | Helen | - | - | - | - | - | - | - | Missing | - | - | - | - | - |
| N-1428/98 - | Helen | - | - | - | n+ | - | N | - | slightly positive | - | - | - | - | - |||  |  |  |  |  |  |  |  |  |  |  |  |  |  |  |
| --- | --- | --- | --- | --- | --- | --- | --- | --- | --- | --- | --- | --- | --- | --- |
| T-1428/98 - | Helen | - | - | - | - | - | - | - | Missing | - | - | - | - | - |
| N-1287/98 | - | Helen | - | - | - | - | - | - | - | Missing | - | - | - | - | - |
| T-1287/98 | - | Helen | - | - | - | nc+ | - | Nc | - | slightly positive | - | - | - | - | - |||  |  |  |  |  |  |  |  |  |  |  |  |  |  |  |  |  |  |  |  |  |  |  |  |  |  |  |  |  |  |  |  |
| --- | --- | --- | --- | --- | --- | --- | --- | --- | --- | --- | --- | --- | --- | --- | --- | --- | --- | --- | --- | --- | --- | --- | --- | --- | --- | --- | --- | --- | --- | --- | --- |
| N-1176/98 | - | Helen | - | - | - | n+ | - | N | - | slightly positive | - | - | - | - | - |||  |  |  |  |  |  |  |  |  |  |  |  |  |  |  |  |
| --- | --- | --- | --- | --- | --- | --- | --- | --- | --- | --- | --- | --- | --- | --- | --- |
| T-1176/98 | - | Helen | - | - | - | nc+ | - | Nc | - | slightly positive | - | - | - | - | - |

  
  
 Tissue MicroArray Common Data Elements
  
  
Specimen\_id 
block specimen identification number  
  
Method\_of\_reading\_data 
Datatype: Character String   
Maximum Occurrence: Unlimited   
Definition: A textual description of how the data was read, which can
provide information regarding whether the data was read by an individual or
individuals (and their names) or was read by an automated fashion.
A description of image analysis hardware, software, manufacturer,
version numbers, and the name of the data manager.
Comment: Attributes are optionally available for this element
Attribute: name is process and key is "automated" or "manual".
  
  
Names\_of\_individuals\_reading\_results 
  
  
Notes 
  
  
No\_of\_cells\_counted 
  
  
 No\_of\_nuclei\_counted 
Datatype: Integer  
Maximum Occurrence: Unlimited   
Definition: This only for FISH, should be an integer.
  
  
 Intensity\_of\_staining 
Datatype: Decimal number   
Maximum Occurrence: Unlimited   
Definition: If the results of staining can be represented by an intensity number, the number can be included in this element.
  
  
 %tissue\_staining 
Datatype: Decimal number ranging from 0 to 100.   
Maximum Occurrence: Unlimited   
Definition: If the results of staining can be represented
by a percentage, then this element can be used to contain the data.
Comment:
  
  
Compartment\_staining 
Datatype: Character String  
Maximum Occurrence: Unlimited  
Definition: If the staining is restricted to particular organelle(s),
they can be listed in this element.  
Comment:
  
  
Staining\_pattern 
Datatype: Character String  
Maximum Occurrence: Unlimited  
Definition: If the staining has an identifiable pattern, then
this information can be included in this element.
Comment: Some values are dot, linear, granular, patternless, diffuse
  
  
Core\_result 
Datatype: Character String  
Maximum Occurrence: Unlimited  
Definition: Empty element that contains the other elements related to\_core\_results  
  
Other\_comments
Datatype: Character String  
Maximum Occurrence: Unlimited  
  
  
Core\_image\_filename 
filename of core\_image
Datatype: Character String   
Maximum Occurrence: Unlimited
  
  
Image\_format 
Datatype: Character String  
Maximum Occurrence: Unlimited  
Definition: Image format  
Comment: Examples are jpeg, tiff, png.
  
  
Image\_magnification 
Datatype: Integer  
Maximum Occurrence: Unlimited  
Definition: This is the magnification at which the image was recorded. This should be as an
integer ranging from 1 to 1000.  
Comment:
  
  
Other\_comments\_on\_images
