# Supplementary material for: TmaDB: a repository for tissue microarray data
Source: BMC Bioinformatics. 2005 Sep 1;6:218. doi: 10.1186/1471-2105-6-218 (PMC1215475; doi:10.1186/1471-2105-6-218)
Supplement: Additional File 1 — This compressed (gz) file contains two directories tmadb_bmc_html and tmadb_bmc and two files, create_tmadb.txt and a README file which can be extracted using gunzip software. The create_tmadb.txt file contains all the MySQL create commands for creating tables contained in the database. The README file provides instructions to help the user install the software. The tmadb_bmc_html directory contains html, xml and text files required for interfacing with the cgi programs. The tmadb_bmc directory contains ten files, nine files with the extension cgi and a file named config.pl. config.pl Contains variables that require modification during installation. colo_form_input.cgi Program to upload colorectal pathology information from the Web form. colo_path_input.cgi Program to upload colorectal pathology information from the Web. core_path.cgi Program to upload specific information relating to each core from the Web. keysearch.cgi Program to query the database using a keyword search or a specific specimen identifier. mysql_search.cgi Program to query the database using MySQL statements. table_contents.cgi Program to display the contents of each table in the database. tma_construct.cgi Program to upload TMA design construct information from the Web. tma_result_input.cgi Program to upload TMA experiment protocol and results from the Web. unknown_path.cgi Program to upload pathology information from the Web for specimens where the diagnosis is unknown. [file 1471-2105-6-218-S1.gz › tmadb/tmadb_bmc_html/unknown_format.htm]

 Submission of pathology data associated with each block specimen on TMA
  
  


|  |  |  |  |  |  |  |  |  |  |  |  |  |  |  |  |  |  |
| --- | --- | --- | --- | --- | --- | --- | --- | --- | --- | --- | --- | --- | --- | --- | --- | --- | --- |
| Specimen\_no/Patient\_code/Path\_no | patient\_code | block\_specimen\_id/LAB\_no | MP\_no/other\_nos | disease\_staus | treatment\_with\_drugs/Previous\_chemo | Response to previous treatments | date\_last seen | tumour\_grade | D.O.B: (yyyy-mm-dd) | Age | Sex | Hospital No | Date of Opn: (yyyy-mm-dd) | Specimen/operation | Site of tumor | Pre-op radiotherapy (yes, no or not known) | Notes |
| 14727/01 | 1 | 9048/99 | 14727/01 | - | Progressive disease,Resistant FU/none Resistant to FU | - | - | - | - | - | - | - | - | - | - | No | - |
| 20313/01 | 2 | 20313/01 | 20313/01 | - | Progressive disease,Resistant FU/none Resistant to FU | - | - | - | - | - | - | - | - | - | - | No | - |
| 23728/96 | 3 | 23728/96 | 23728/96 | - | Progressive disease,Resistant FU/none Resistant to FU | - | - | - | - | - | - | - | - | - | - | No | - |
 5317/00 | 4 | 5317/00 | 5317/00 | - | Progressive disease,Resistant FU/none Resistant to FU | - | - | - | - | - | - | - | - | - | - | No | - |
